# Virtual Light Sensing Technology for Fast Calculation of Daylight Autonomy Metrics †

**DOI:** 10.3390/s23042255

**Published:** 2023-02-17

**Authors:** Sergey Ershov, Vadim Sokolov, Vladimir Galaktionov, Alexey Voloboy

**Affiliations:** 1 Keldysh Institute of Applied Math RAS, 125047 Moscow, Russia; 2 Faculty of Software Engineering and Computer Systems, ITMO University, 197101 St. Petersburg, Russia

**Keywords:** virtual light sensing technology, lighting simulation, Daylight Autonomy, spatial Daylight Autonomy, Annual Sunlight Exposure, blinds control

## Abstract

Virtual sensing technology uses mathematical calculations instead of natural measurements when the latter are too difficult or expensive. Nowadays, application of virtual light sensing technology becomes almost mandatory for daylight analysis at the stage of architectural project development. Daylight Autonomy metrics should be calculated multiple times during the project. A properly designed building can reduce the necessity of artificial lighting, thus saving energy. There are two main daylight performance metrics: Spatial Daylight Autonomy (sDA) and Annual Sunlight Exposure (ASE). To obtain their values, we have to simulate global illumination for every hour of the year. A light simulation method should therefore be as efficient as possible for processing complex building models. In this paper we present a method for fast calculation of Daylight Autonomy metrics, allowing them to be calculated within a reasonable timescale. We compared our method with straightforward calculations and other existing solutions. This comparison demonstrates good agreement; this proves sufficient accuracy and higher efficiency of the method. Our method also contains an original algorithm for the automatic setting of the sensing area. The sDA metric is calculated considering blinds control, which should open or close them depending on overexposure to direct sunlight. Thus, we developed an optimization procedure to determine the blinds configuration at any time.

## 1. Introduction

Virtual sensing technology uses mathematical calculations instead of natural measurements when the latter are too difficult or expensive. This technology has been successfully developed and used over the past three decades in various fields, both to expand the capabilities of a number of real sensors and to develop new sensing technologies [1,2]. Nowadays, virtual sensing technologies can be reinforced with a machine learning approach for the development of methods and solutions that can provide a high level of quality monitoring with minimal hardware and cost [3,4].

The widespread use of virtual and real sensors, particularly light sensors, in building construction, interior design and smart home solutions will allow them to achieve a level of optimization and improvement that was not previously considered economically viable. The tasks which can be solved with the help of this technology are not limited to the human environment. For example, they also help to solve the agricultural problems of increasing yields in greenhouses or mixed (indoor/outdoor) plants [5]. If, during the building operation, virtual sensing technologies allow expansion of the scope and increase efficiency of real sensors, then during the design of a building that virtual sensing technology remains the only applicable means, because the building simply does not yet exist. Light sensors, which make it possible to analyze the illumination of premises, play an important role in the energy-saving design and operation of buildings. Modern building design requires illumination analysis of premises and imposes a green building certification program called Leadership in Energy and Environmental Design (LEED) [6].

The spatial Daylight Autonomy (sDA) and Annual Sunlight Exposure (ASE) are the two widely used metrics from the LEED program. These characteristics are explained in detail in the IES LM-83-12 standard [7] (IES—Illuminating Engineering Society) but can be described briefly as follows:

- Spatial Daylight Autonomy (sDA) is the metric describing the annual sufficiency of ambient daylight levels in interior environments. It is the fraction of the analysis area where the daylight is above 300 lx for more than 50% of the annual observation period. This value is denoted (sDA_300,50%_); the 300 lx and 50% are parameters and may vary.
- Annual Sunlight Exposure (ASE) is the metric that describes the potential for visual discomfort in an interior work environment. It is defined as the percentage of an analysis area where direct sunlight illuminance is above 1000 lx for more than 250 h per year. This value is denoted (ASE_1000,250h_); the 1000 lx and 250 h are parameters and may vary.

These metrics allow estimation of the design quality of the examined object (say, a hall or room in the building), i.e., roughly, whether the windows are large enough, whether they open to the right side, and so on [8]. The higher the ASE value, the stronger the discomfort from overexposure. Typically, the acceptable level of ASE is below 10%. The situation with the sDA metric is the opposite: the higher the better, because less artificial light is needed, which decreases energy consumption. These two metrics are intended to be applied to workspaces of similar purposes, such as open offices, classrooms, conference and multi-purpose auditoriums, and lobbies. For this type of premises, modern architectural solutions often use large areas of glass and blinds. The positional design of the blinds and the shading they provide greatly affects the distribution of daylight in a room.

To obtain the sDA/ASE values, we need to measure the illuminance distribution in the room every hour throughout the year. Some studies suggest calculating metrics not for the whole year, but for seasonal periods [9]. Should the building already exist, it can be obtained by a grid of real sensors. However, if the building is just being designed, it is only possible to represent a set of light sensors with the sensing working plane and calculate spatial distribution of illuminance. A similar approach was used, for example, in [10]. Simulation of ASE calculates illuminance from direct sunlight only, ignoring skylights and interreflections. Simulation of sDA calculates full illumination (direct and indirect, sun and sky). The lighting engine used for calculations must therefore separate these components.

There are currently about 40 different programs that allow you to calculate metrics and indicators of natural light for architectural projects [11], such as DesignBuilder [12], DIVA for Rhino (integrated in Climate Studio now) [13] and DL-Light [14]. Most of them use Radiance [15,16] as the lighting simulation engine. On one hand, the use of the Radiance kernel solves the problem of reliability of simulation results, since this software has been repeatedly tested and its physical accuracy is well known. On the other hand, this is a general lighting simulation engine. It is not optimized for multiple simulations of a particular scene for more than 4000 lighting conditions. Therefore, calculation of Daylight Autonomy metrics needs significant resources and is rather time-consuming for complex building models.

Since calculation of the DA metrics is important for architects in their everyday work, ways to speed up and optimize their calculations are continually being proposed. One approach suggests the use of statistical data to quickly obtain values based on the parameters of buildings of the same type (atriums) [17]. Of course, these values will be very approximate and can only be used as a fast estimate for the project draft. Recently, the use of machine learning (ML) technologies for assessing daylight performance in buildings has become popular [18,19,20,21,22,23]. Some studies have also considered the task of computing sDA and ASE metrics for certain types of room and certain designs of exterior façade, and most studies have used the direct modeling variables (e.g., window size, room size) as input parameters [24]. This can seriously limit the applicability of ML models in practical design.

Thus, we focused our study on developing a method for fast and accurate calculation of the sDA and ASE metrics. On one hand, architectural design models are becoming more and more detailed. Models can be arbitrary and may not always be represented by parametric models. When the corresponding virtual scenes turn out to be huge, lighting simulation requires a significant amount of time. On the other hand, architects must constantly monitor compliance with standards, which leads to the calculation of DA metrics, sometimes several times a day. Therefore, it would be desirable to reduce the time of their calculation to minutes, or tens of minutes, on a conventional computer. At the same time, the accuracy of the calculated metrics should be high. We set ourselves the goal of developing methods and algorithms that solve these problems. Our work therefore contributes to (1) development of a fast method for simulation of the direct and indirect illumination components from daylight, (2) automatic specification of the sensing area and (3) elaboration of optimizing the blinds control algorithm. Additionally, lighting simulation engine validation of the Lumicept software [25,26,27,28] against the CIE 171:2006 test suite [29] (CIE—Commission Internationale de l’Eclairage, or International Commission on Illumination) can be considered a supplementary contribution.

## 2. Automatic Specification of Sensing Area

Normally, the analysis area—i.e., the sensing domain—is part of the horizontal plane, elevated some 76 cm above the floor and offset from the walls by some 30.5 cm. It is where we must calculate (or measure) illuminance distribution, so we cover it with a grid of sensors (natural or virtual) [10]. A cell of that grid is a virtual sensor, thus will be termed a sensing cell. Parameters of the subdivision (space grid) of the analysis area for ASE and sDA calculation are regulated by the IES LM-83-12 requirements [7]. However, the maximum size of the grid cell, elevation above the floor and offset from the wall(s) are variable and have to be specified. The manual specification of such a grid may be inconvenient because the analysis area can have a complex, not rectangular, shape. To solve this problem, a special method of automatic grid definition has been elaborated. It requires the geometry of the sensing area to be subdivided into separate parts, with an individual grid then constructed for each part.

The architect needs to specify only three parameters and the link to the part (ground part, typically floor) above which the illumination grid should be placed. These parameters are:

1. “Cell size”, defining the maximum size of the grid cell (if the exact value is inaccessible for an integer number of cells, the nearest smaller size is adopted);
2. “Offset”, specifying the gap between the grid and the walls (gap between the edges of the grid and the boundaries of the linked part);
3. “Elevation”, defining the vertical distance between the floor plane and the grid (the vertical position of the working plane).

**Figure 1:**
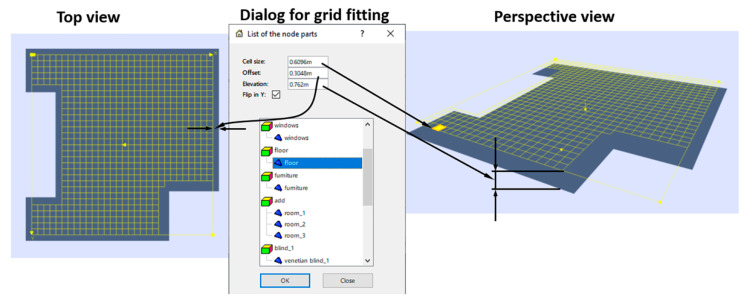
Generation of the illuminating grid.

The default values of these parameters are specified according to the Illuminating Engineering Society (IES) recommendations. In Figure 1 the yellow grid is created with the fitting procedure.

The fitting procedure is rather simple. First, we determine the plane using the covariation basis (in particular, the Karhunen–Loève basis [30]), i.e., eigenvectors of the covariation matrix:

$$C_{i,j}=\frac{1}{N}\overset{N}{\underset{k=1}{\sum }}x_{k,i}x_{k,j}-\left(\frac{1}{N}\overset{N}{\underset{k=1}{\sum }}x_{k,i}\right)\left(\frac{1}{N}\overset{N}{\underset{k=1}{\sum }}x_{k,j}\right)$$

where $x_{k,i}$ is the *i*-th coordinate of the *k*-th point (vertex) of the scene part P this sensing grid is fit to. The eigenvector corresponding to the smallest eigenvalue is the normal to the plane *n*. The sign of its orientation is determined so that its dot product with the zenith direction is positive. If deviation from the zenith exceeds some threshold, a warning is issued that the analysis area is too inclined. Now equation of the plane is (x⋅n)=d where $d=\frac{1}{N}\overset{N}{\underset{k=1}{\sum }}\;(x_{k}\cdot n)+E_{\;}$, and the elevation *E* is 76 cm by default (corresponding to the LEED standard) and can be manually varied.

The next step is to find orientation of the grid lines within this plane, i.e., direction of the axes of the local (tangent) coordinate system (*u*, *v*) in that plane. This is a fast procedure, thus it is implemented via a simple linear search of the angle of rotation about the normal plane. The criterion is that the bounding rectangle (oriented along the local axis) of projection of P (i.e., of $\left\{x_{k}\right\}$) has a minimal area.

After that, this bounding box (rectangular analysis area in the new coordinates) is subdivided by a rectangular grid into equal cells. The cells must be separated by the given offset from the boundaries of the scene part projection onto the plane. To this end, we first calculate rasterization of that projection by tracing rays along the normal plane and seeing whether they hit or miss the scene part. According to our experiments the resolution about 1000 along the larger size is enough; along the short size it is chosen to have square pixels. The pixels inside the projection have value 0 and the rest (outside ones) are 1. The boundary pixels are those which keep 0 themselves, while there is an adjacent one that keeps 1. After separating all boundary pixels, we cycle over them and “blur” by drawing a circle of the offset radius with the center in each boundary pixel and setting the value to, say, 2 for pixels in that circle. Pixels that are closer than the offset to the boundaries then have value 1 or 2 and only those with value 0 are “internal”.

We calculate the bounding rectangle of these “internal” pixels and cover it with the rectangular grid of cells. The grid resolution is chosen as the smallest integer for which cell size is below the desired value. Since the “interior” may have a complex shape, some cells of its bounding rectangle can be outside the inner area. We mark the cells which do not contain “inner” pixels as “disabled”. They will not be used in illumination calculation. An example of a generated grid is presented in Figure 1.

## 3. Methods of Daylight Simulation for sDA and ASE Calculation

The proper daylight model for the correct and precise calculation of the sDA and ASE characteristics should be based on the Perez sky model. The Perez formulas of the sky luminance distribution (i.e., the sky goniogram) can be found in [31,32] and our simulations are based on them.

There are several main parameters of the Perez sky model. The first part is related to the sun position (sun azimuth and elevation angles). The definition of these parameters is well known [33,34]. They can be calculated from the geographic location and the specific date and time. The next portion of parameters is related to the values measured at the particular date and time: the Direct Normal Illuminance (DNI) and Diffuse Horizontal Illuminance (DHI). These data are provided with meteorological stations around the world and are part of the Typical Meteorological Year (TMY). We use the EnergyPlus Weather (EPW) format; the data are available on the Internet [35]. The DNI and DHI values from the EPW file are directly used in Perez’s sky goniogram. Note that these values are given in radiometric units, while the Perez formulas operate photometric ones. Fortunately, the EPW file has an additional set of DNI and DHI in photometric units. It is used as the scale factor for the radiometric to photometric conversion.

The ASE/sDA metrics can be calculated in a straightforward way according to their definitions by Forward Monte Carlo ray tracing (FMCRT) [36]. FMCRT is an accurate method, though not fast; thus, straightforward calculations require significant calculation time. Calculation of ASE/sDA values with FMCRT can be done as follows. For each target time moment we calculate:

1. The direct sun illumination (to be used for ASE). Reflection of all scene surfaces is therefore set to 0 to exclude secondary illumination. Skylight is also turned off. All blinds are open (as required for ASE);
2. Full illuminance (direct and indirect, sunlight and skylight) for the configuration of blinds exactly as in our method. Skylight is turned on and surface reflectance is set to the values specified for the scene.

We then calculate the ASE and sDA metrics from the time series of illuminance distributions. All FMCRT calculations should be run with high accuracy to avoid stochastic noise influence.

We propose a method for calculating DA metrics which is much faster than the straightforward one, but has similar high accuracy.

### 3.1. Calculation of Direct Sunlight Component

ASE is calculated on the base of illuminance created with direct sunlight, so the ray does not change its direction. Attenuation (for example, by a tinted glass) is taken into account. Illumination of a sensing cell is calculated as follows: we take a random point in this cell, then from it we fire the ray “towards the sun”. If the ray undergoes reflection or diffuse scattering, this ray makes no contribution. If it undergoes only specular transmission or no event, its contribution is equal to the attenuation factor. Since different rays (points) are independent, accuracy of the estimated illuminance can be taken from the sample variance. When the error drops below the desired tolerance, we go to the next cell, etc. The calculation applies for all target time moments (i.e., annually with 1 h step), skipping only those when the sun is below the horizon. We do not use “interpolation” like we do for indirect sunlight (see below) for better accuracy, and also because this part of the calculations is relatively fast.

The sDA calculation is based on the full illumination, including light scattered diffusively. Such calculation can take significant time. The whole annual period consists of 365 days and a dozen calculations have to be run for each day. Thus about 4000 calculations should be run for the annual result. To accelerate sDA calculation, several approximations are used.

### 3.2. Calculation of Indirect Sunlight Component

**Figure 2:**
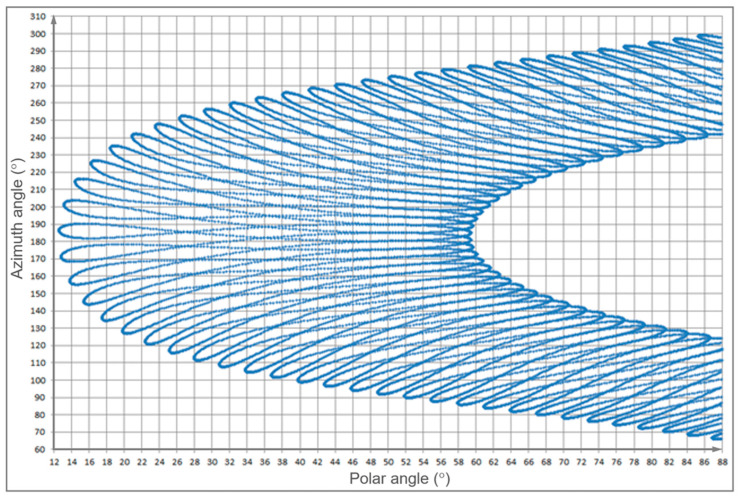
Sun position in the hemisphere during a year; azimuth is vertical and polar angle is horizontal.

This is calculated with the classical Forward Monte Carlo ray tracing [36], ignoring the direct rays which had already being counted, as described in Section 3.1. Indirect illumination is not very sensitive to the sun direction. This allows the amount of calculations to be reduced: unlike for direct sunlight, here we make calculations not for all target time moments but for distinct sun positions. Namely, we first collect the set of all sun positions above the horizon. For an annual period, the polar angles of the sun form a spiral (Figure 2).

We then take a Klems grid [37] (subdivision of the hemisphere into approximately equal square cells) of 31 cells in polar angles and a corresponding (to make them quadratic) number in azimuth for each polar angle. In total there are about 1000 cells and 1000 vertices. This reduces the number of calculations by about four times compared to processing all target time moments.

Now we calculate illuminance for a parallel light source with unit flux, each of these in about 1000 directions (vertices of the Klems grid). To be precise, we calculate only the vertices of those cells which contain at least one of the target sun positions (Figure 2). This reduces their number to about 600. Then for each target sun position we can estimate illuminance as follows. We take a cell of the Klems grid which the target sun point belongs to. Illuminance of each sensing cell for the target sun position is bi-linear interpolation over the four “bracketing” directions times the sun flux (and color) at the target moment. We should do this for all target time moments, for all sensing cells.

### 3.3. Calculation of Skylight Component

This is also calculated with the FMCRT, though not for all target sun configurations, sing a sort of “interpolation” as is done for indirect sunlight.

Illumination by the sky is determined by the skylight goniogram. Due to the linearity of the problem, illumination of a sensing cell is a linear functional over the goniogram. In case the goniogram is a tabulated function, this functional has a form like

(1) $$I_{i}=\underset{j}{\sum }R_{i,j}L_{j}$$

where *i* is the index of the sensing cell and *I_i_* is the target illumination of this cell, *j* is the index of the goniogram vertex, $L_{j}$ is sky luminance in this vertex and $R_{i,j}$ is “the response function”, i.e., illumination of the *i*-th sensing cell from the sky goniogram which is 0 in all vertices but the *j*-th one where it is 1.

To compute the matrix $\left\{R_{i,j}\right\}$ we therefore cycle over all the sky goniogram vertices, setting 1 for this vertex and 0 otherwise, then run the classical FMCRT and calculate illumination in all cells. After that, for each target moment we calculate the sky goniogram from the Perez model, and apply (1) for each sensing cell without any expensive ray tracing. Obviously the response function calculation requires time proportional to the number of sky goniogram vertices, thus one needs to reduce its resolution as much as possible. Meanwhile, for an accurate representation of the sky goniogram, its resolution must be as high as possible. From experience, a due compromise is to use a Klems grid of about 150 vertices.

## 4. Calculation of Illumination with Blinds

The calculation of sDA metrics requires support of the blinds control. If the illuminance distribution meets the overexposure condition, some blinds have to be closed and this state is to be used in the sDA calculation. The default overexposure condition is that more than 2% of the analysis area has illuminance greater than 1000 lx under direct sunlight. Usually there are several blinds which can be opened or closed independently. Blinds may shade only part of the window area, so cannot block the light completely. Any blind can be in either an open or closed state. We do not consider gradual shadowing. Closing all the blinds solves the problem of overexposure; however, it becomes too dark in the rooms because most of the light is blocked. Opening all of them lets the light in but, possibly, at the expense of discomfort from overexposure.

We must therefore find their optimal configuration (only some blinds have to be closed) that provides the best daylight illumination: as high illumination as possible but without overexposure from direct sunlight. This is obviously achieved by closing the fewest blinds possible. The state of blinds is calculated independently for each target time moment from direct sunlight, ignoring the rest of the components.

### 4.1. What Blinds to Close

We have several blind groups, all enumerated by index *k*. First, we open all blinds. We then try to close just one of them: maybe this will be enough. We cycle over all the blinds, denoting their index as *k,* and close only the *k*-th blind, while we open all the rest. In each case we calculate illumination under direct sunlight $I^{\left(k\right)}$ as described in Section 3.1 and calculate the total area of all sensing cells, where illuminance > 1000 lux. $f_{k}$ is the ratio of that area to the area of all cells. If $f_{k}\leq 2\%$ for some *k* then it is enough to close just the *k*-th blind and we have found the optimal configuration for this time moment.

If the condition $f_{k}\leq 2\%$ had never been satisfied, the procedure finishes, giving us arrays of $\left\{f_{k}\right\}$ and $\left\{I^{\left(k\right)}\right\}$, where $f_{k}$ is the overexposed fraction and $I^{\left(k\right)}$ is direct sunlight illumination when only the *k*-th blind is closed. In this case, closing any single blind group is not enough and we have to close at least two of them. So now we try to close two or, if this did not help, three, four, etc. groups of blinds.

The number of possible combinations is very large, but happily, one can instantly calculate an illuminance table for any combination of blinds from $\left\{I^{\left(k\right)}\right\}$ obtained above. It does not require expensive ray tracing; only summation/subtraction of illuminance tables. Then from this illuminance table we calculate the overexposure fraction *f*.

We search for a combination of two blinds closing which will be enough (for the overexposure fraction to drop below 2%). A natural choice is that the first blind is the “most efficient” one, i.e., that with the smallest $f_{k}$. We then must try to choose only the second blind. When the overexposure fraction *f* drops below 2%, we adopt the current configuration of blinds as the optimal one and finish.

If this never happens for any second blind, then two blinds are not enough. We then try to close three blinds at a time. This time we close the two “most efficient” blinds, i.e., those for the two smallest $f_{k}$ in the array. It is then enough to search for the third blind to close, which is again done by cycling over all of them. When the overexposure fraction *f* drops below 2%, we adopt the current configuration of blinds as the optimal one and finish.

Otherwise, we must try to close four blinds at a time; if this was not enough, then five, and so on. If the overexposure fraction is above 2% even for all blinds closed, then the optimal configuration is “all blinds closed”.

### 4.2. Fast Calculation of Blinds Effect

As explained above, for *N* blinds there are about $2^{N}$ different combinations of open/closed blinds. For each of them we must calculate illumination and the overexposed area fraction. Meanwhile, direct calculation of illuminance by ray tracing can be very expensive. Happily, utilizing the linearity of the illumination problem, it is enough to calculate only *N* combinations (only one blind is closed, with the remainder open). Indeed, illuminance of a sensing cell is the average over these rays from the cell to the sun:

(2) $$I=const\times \left(\underset{through\;E}{\underset{︸}{\underset{i}{\sum }{ℐ}_{i}}}+\underset{through\;B_{1}}{\underset{︸}{\underset{i}{\sum }\tau_{i}^{\left(1\right)}{ℐ}_{i}}}+\underset{through\;B_{2}}{\underset{︸}{\underset{i}{\sum }\tau_{i}^{\left(2\right)}{ℐ}_{i}}}+\underset{through\;B_{3}}{\underset{︸}{\underset{i}{\sum }\tau_{i}^{\left(3\right)}{ℐ}_{i}}}+\cdots \right)$$

where E is the domain which cannot be blinded (i.e., it is outside of all blinds), $B_{k}$ is the *k*-th blind, *i* is the index of ray, ${ℐ}_{i}$ is the contribution of this ray to average cell illuminance and $\tau_{i}^{\left(k\right)}$ is attenuation for the *i*-th ray through the *k*-th blind area. It can be written as

$$\tau_{i}^{\left(k\right)}=\left(1-\chi_{k}\right)\tau_{i}^{\left(o\right)}+\chi_{k}\tau_{i}^{\left(c\right)}$$

where $\chi_{k}=1$ when the *k*-th blind is closed and $\chi_{k}=0$, otherwise $\tau_{i}^{\left(o\right)}$ is attenuation when the blind is open and $\tau_{i}^{\left(c\right)}$ is attenuation when this blind is closed.

The expression (2) can be identically rewritten as

$$I=I^{\left(open\right)}-\underset{k}{\sum }\chi_{k}\left(I^{\left(open\right)}-I^{\left(k\right)}\right)$$

where $I^{\left(open\right)}$ is illuminance when all the blinds are open and $I^{\left(k\right)}$ is illuminance when all blinds are open but the *k*-th one is closed. Therefore, we can instantly calculate illumination for an arbitrary configuration of blinds (determined by the set of $\left\{\chi_{k}\right\}$) if we know illumination for the “base” configurations when only one blind is closed. The $I^{\left(open\right)}$ and $I^{\left(k\right)}$ can be illuminance of a particular sensing cell or can be illuminance tables (matrices for all cells).

The difficulty is that illumination is calculated by Monte Carlo integration, thus is noisy. Subtracting two close, while noisy, illumination values may give a negative or at least inaccurate difference. Therefore, this procedure requires that $I^{\left(open\right)}$ and all $I^{\left(k\right)}$ be calculated with accuracy much higher than for the rest of the calculations. We used a 0.25% accuracy level here.

### 4.3. Illumination for Arbitrary Blinds Configuration

The full illuminance is the sum of three components of light, which are calculated differently.

#### 4.3.1. Direct Sunlight Component

After completion of the blinds control phase, we know the blinds configuration (which are open and which are closed) for all time moments. Since this is based upon the overexposed area fraction, we also calculate illuminance under direct sunlight for all sensing cells. Since calculations began with an “all blinds open” state, we know this illuminance too, and can already compute the ASE metrics. The two remaining illumination components—full skylight and indirect sunlight—must also be calculated for the found state of blinds. It is not trivial because these calculations are not performed at the target time moments (when we know the blinds configuration) but for the set of sun/sky states from which illumination for sun position and sky goniogram for the target model are interpolated.

#### 4.3.2. Skylight Component

Skylight illumination is calculated from the response matrix $\left\{R_{i,j}\right\}$; see Equation (1). Its element is illuminance of the *i*-th sensing cell when the luminance of sky is 0 at all vertices but the *j*-th one where it is 1. This illuminance naturally depends on which blinds are closed; that is, the response matrix depends on the blind state. We must thus calculate it for any blind state which is ever used (i.e., for the target time moment). We first cycle over all the time moments and gather all the different (because several time moments may use the same state of blinds) configurations of blinds. For each, we then calculate the response matrix as described in Section 3.3.

#### 4.3.3. Indirect Sunlight Component

The indirect sunlight component is calculated similarly to the skylight (Section 3.2). We use a Klems grid and calculate illuminance for a parallel illumination (with unit flux), with a direction equal to the vertex. Afterwards, we cycle over all target time moments; for each we find four directions from that grid that bracket the sun position at this time moment. The target illumination is then the weighted sum over illuminations for these four directions, with weights the same as when interpolating the target sun position and times the target sun flux as described in Section 3.2. Each direction of the grid can now be used at several target time moments, thus we must calculate illumination from it for all target time moment blinds configurations that use this direction. Usually there are not many, because the grid of directions is rather dense and so each grid cell does not contain many target moments. Blind state can be the same for different time moments and we must select all the different states. We then compute illuminance of the cell for the parallel illumination, with unit flux and direction given by the chosen vertex. This calculation is done for all different blind states. From experience, there are few for most vertices, much less than the total number of different blind states throughout all time moments. This also reduces the amount of calculations.

We then cycle over all target time moments; for each, we find the four grid directions that bracket it. We then go through the array of blind states stored in each of them, and if the current one is different, we add it to the set. After completion, for each grid direction we have all the blind states needed for it. Then we cycle over each direction, skipping those not used for any target time moment, and calculate illumination for a unit parallel illumination from that direction for all blinds configurations saved at this grid vertex.

Eventually we cycle over the target time moments; for each, we find the four bracketing directions and take their weighted sum of illuminance calculated as the target blinds configuration.

To obtain the DA metrics we combine the calculated data. Full illumination is the sum of illumination under the target blinds configuration by the direct sunlight component, indirect sunlight component and skylight component. It is calculated for all time moments and the resultant array is used to calculate sDA. The direct sunlight illumination under all blinds open is used to calculate ASE.

## 5. Results

### 5.1. Verification Scenes

To verify the quality and efficiency of sDA/ASE calculation, three scenes have been used.

**Figure 3:**
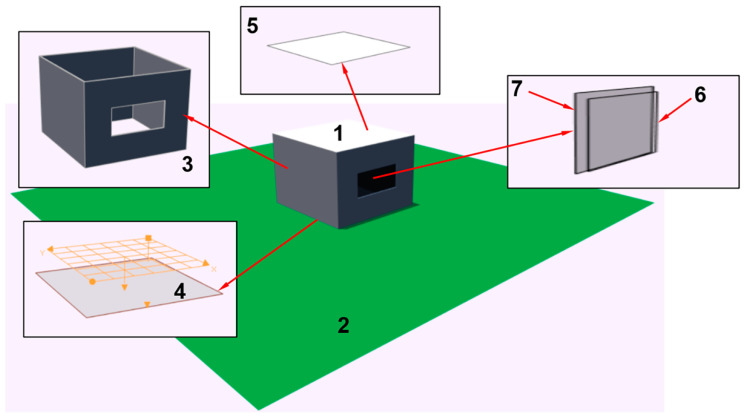
Scheme of the first verification scene.

The first scene (Figure 3) is the most trivial. It is close to the Commission Internationale de l’Eclairage (CIE) tests used for validation of our approach and the Lumicept [26,28] lighting simulation engine in Section 6. The model consists of a box *1* with sizes 4 m × 4 m × 3 m placed on the ground plane *2*. The geometry of the box includes walls *3*, a floor *4* and ceiling; see Figure 3. One of the walls *3* has the opening. The opening is closed with window—a transparent plane *6*. Blinds are represented as a plane *7* (Figure 3). All surfaces of the box and the ground plane have diffuse reflectance: 50% for the walls, 30% for the floor, 70% for the ceiling and 20% for the ground plane. The window has specular transparency of 95% and the blinds have specular transparency of 20%. The sensing plane grid used for calculating sDA/ASE has parameters recommended in IES LM-83-13. It is placed 76 cm above the floor; the offset from the walls is 30.5 cm and the cell size is not greater than 61 cm; see *4* in Figure 3.

**Figure 4:**
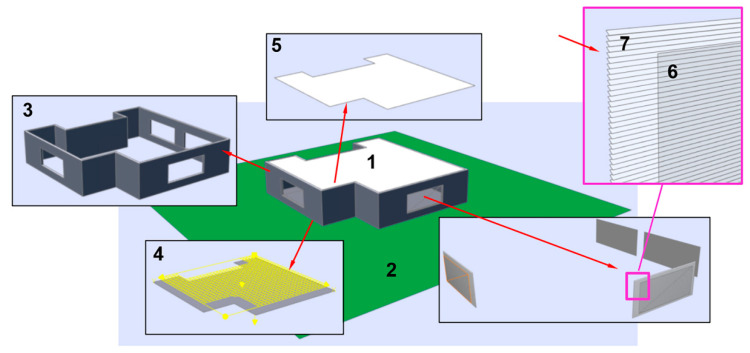
Scheme of the second verification scene.

The second scene has more complex geometry. The scene model is presented in Figure 4 and consists of the building *1* and the ground plane *2;* see Figure 4. The windows *6* have Venetian blinds *7*. The wall surfaces, floor *4* and ceiling have diffuse reflectance of 50%, 20% and 70%, respectively. The optical properties and illumination grid are set according to the Illuminating Engineering Society (IES) recommendations. The blinds are modeled more realistically than in the first scene. They are similar to “Venetian blinds” and subdivided into three groups: each wall with its window and blinds forms a separate blind group.

**Figure 5:**
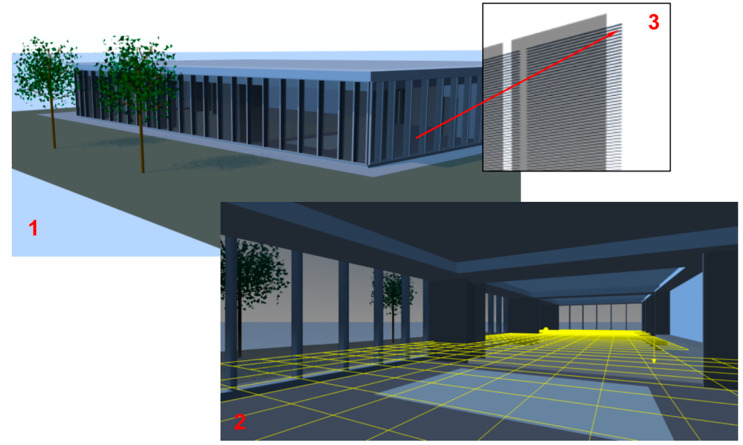
The third verification scene.

The third scene presents a real hall with complex geometry (Figure 5, 1—outdoor view, 2—indoor view). The blinds (3 in Figure 5) form three independent groups as in the second scene.

In all these examples, the Perez sky model was used for simulation and the TMY file was taken from the EnergyPlus dataset [35]. Most simulation parameters correspond to the IES standards for ASE/sDA calculation. The entire annual period was covered, from 1 January to 31 December, 08:00 to 18:00 each day, with a one-hour step. Simulation accuracy was set to 5%. Blinds are closed automatically according to the standard values of overexposure (2% area with ≥ 1000 lx).

Table 1 shows Daylight Autonomy results for all three verification scenes for the entire annual period. Blinds control was not activated in these simulations. Calculation time in this and other tables is for PC, Intel(R) Core(TM) i9-9880H CPU @ 2.30GHz, 8 Core(s), 16 Logical Processor(s), 16GB RAM, Microsoft Windows 10.

Table 2 shows Daylight Autonomy results for all three verification scenes for the entire annual period with blinds control. The criteria to open/close blinds were the same in all simulation examples: direct illuminance threshold = 1000 lx and area fraction = 2%.

The ASE values are the same in both tables because the metric is calculated for all blinds open. The sDA values are lower in Table 2 because this metric is calculated with blinds control. Additionally, calculation time increases with blinds control.

### 5.2. Comparison with Accurate Lighting Simulation

We also verified our accelerated method, comparing its results with those calculated by the Lumicept [28] lighting simulation engine based on Forward Monte Carlo ray tracing. FMCRT was used according to the scheme described in Section 3. To keep calculation time in reasonable bounds, we used a rather short simulation period (5 days). To increase reliability of verification, we took four such time periods (5 days in winter, summer, autumn and spring). The FMCRT error was set at less than 0.25% to avoid stochastic noise influence and provide high accuracy of results. Configuration of blinds for each target time moment was taken exactly as in our accelerated method.

Table 3, Table 4 and Table 5 show DA metrics, calculation time and errors for the first, second and third verification scenes calculated by our method and by FMCRT. Error is the relative difference between the values calculated in a straightforward way and by our fast method.

As is seen in Table 3, Table 4 and Table 5, the results of DA metric calculations are very close to our method, without any tricks or interpolation techniques for the accurate lighting simulation. The difference is so low because they compare “threshold-based” values like ASE and sDA metrics. Roughly, these metrics relate to the count of sensing cells where illuminance is above or below some threshold. Thus, a change of illuminance, unless it moves the value across that threshold, has absolutely no effect on sDA and ASE.

**Figure 6:**
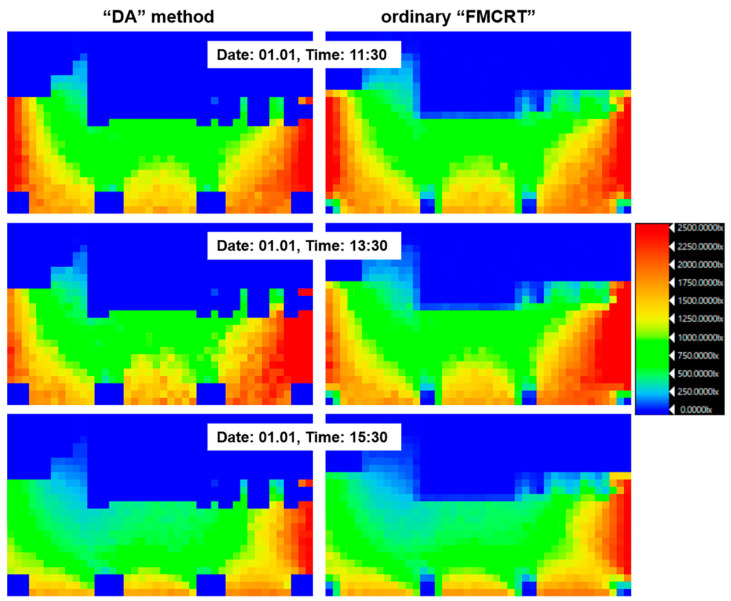
Illuminance distribution for the third verification scene calculated by our (“DA”) method and by FMCRT.

Figure 6 presents several hourly snapshots of illuminance distribution calculated by our method and FMCRT. We can see the illuminance distribution is close as well.

Table 6 shows the calculation time of our method and of FMCRT for different simulation periods. We can see that the increase in the calculation time of our method is very moderate, while the FMCRT calculation time increases linearly with the number of days.

It can be easily calculated that about 30 h will be needed for the FMCRT to calculate annual ASE/sDA metrics. Our method takes only half an hour to do this (Table 2).

### 5.3. Comparison with Existing Solutions

As mentioned earlier, most existing daylight simulation programs are based on the Radiance engine. Many of them are implemented as an extension to the well-known 3D modelers like SketchUp and RhinoCeros. DL-Light produced by De Luminae [14] was selected for the comparison because it uses Radiance for daylight simulation and a simple 3D modeler SketchUp oriented for architectural modeling.

**Figure 7:**
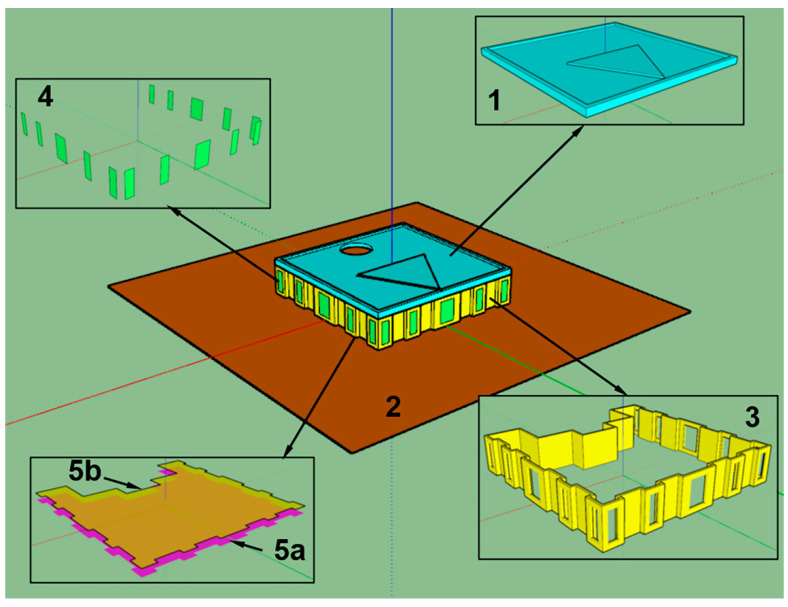
SketchUp model for comparison with DL-Light.

**Figure 8:**
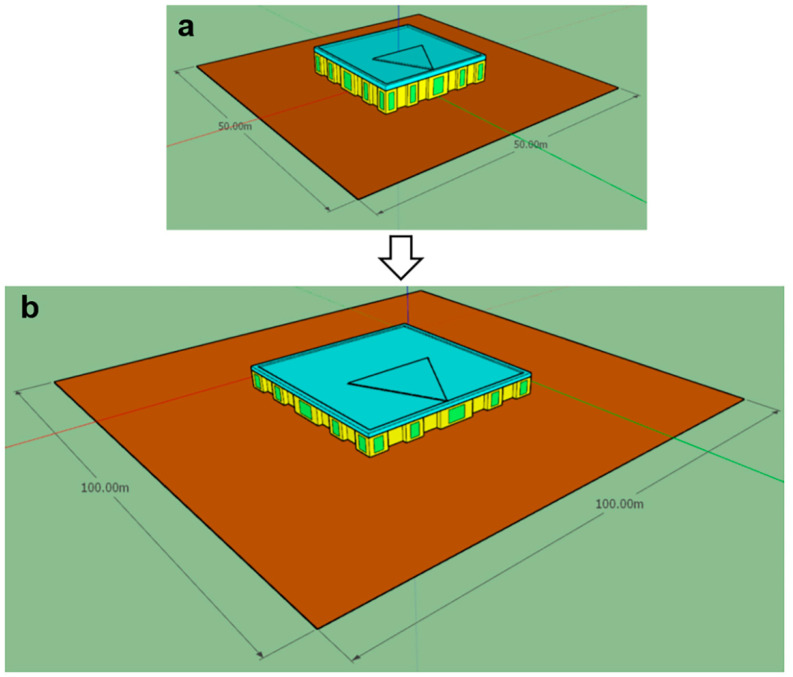
Two variants of model: (**a**) initial and (**b**) scaled.

We prepared two building models (Figure 7 and Figure 8) for comparison.

The first model (Figure 7) consists of ceiling *1*, ground plane *2*, walls *3*, windows *4* and floor *5a*. Illuminance is calculated over the working plane *5b* elevated 76 cm above the floor (default for sDA/ASE standard). The optical properties are as follows: reflectance is 70% for the ceiling, 50% for the walls, 30% for the floor and 20% for the ground plane. The windows are transparent surfaces with transmittance of 91.8%. The second model was prepared by scaling the first to complicate it (Figure 8). Scaling increases the number of sensors (i.e., the cells where illumination is collected), which can result in a decreased calculation speed.

Table 7 shows sDA/ASE simulation results for both models A and B.

From Table 7 we can see that the ASE/sDA metrics are rather close (save for ASE values for model B; this difference is discussed below). The calculation speed for initial model A is also similar, while our method is slightly faster. However, in the case of more complex model B, DL-Light is significantly slower.

## 6. Validation of our Daylight Simulation

Our method demonstrates good accuracy in comparison with the straightforward FMCRT simulation by Lumicept (Table 3, Table 4, Table 5 and Table 6). While accuracy of Radiance has been investigated and reported many times [38,39,40], accuracy of the Lumicept lighting simulation engine needs to be verified. This has been done with the set of CIE 171:2006 tests [29] for validation of lighting simulation software. This set contains many tests. We performed a full validation of Lumicept but present here only the results of test 5.11, the scheme of which is close to the task of DA metric calculation.

**Figure 9:**
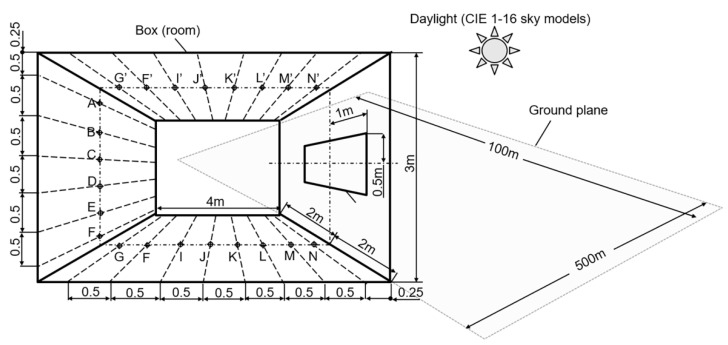
The scheme of scene for test 5.11 from CIE 171:2006.

The 5.11 test (whose scheme is shown in Figure 9) verifies indoor illuminance in the set of points on the floor, wall and ceiling of the room (box). The source of illumination is daylight passing through the opening in the right wall directly or after reflection by the ground plane. Sixteen standard models of (Commission Internationale de l’Eclairage, or International Commission on Illumination (CIE) sky goniogram have been tested. The test is rather complex for simulation software because the ground plane size is not defined, therefore it should be sufficiently large so as not to affect simulation output.

**Figure 10:**
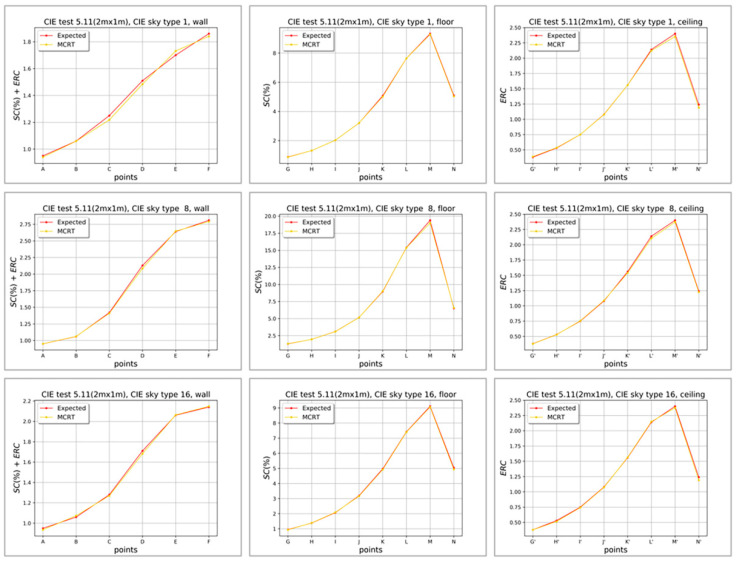
Results of test 5.11 from CIE 171:2006. The yellow line represents the result of Lumicept software (Forward Monte Carlo ray tracing) and the red line is the reference result specified in CIE 171: 2006.

Figure 10 presents the results of simulation in the form of plots for three (of 16) CIE sky models. The output presented for three CIE sky models is as follows: model 1—the first row, model 8—the second row and model 16—the third row. The yellow line is the result of Lumicept software (Forward Monte Carlo ray tracing) and the red line is the reference result specified in CIE 171: 2006. The numerical output for these CIE skylight models is presented in Table 8, Table 9 and Table 10.

Table 8, Table 9 and Table 10 present the results of test 5.11 for three sky types only. We do not present the results for all sky types here, due to their volume; however, the results of testing are very similar. FMCRT shows good agreement with the reference CIE data and the difference (Error) does not exceed 1–2%. Thus, it can be concluded that the Lumicept lighting simulation engine (FMCRT) can be used for the verification of our Daylight Autonomy calculation method.

In Table 7 we see a rather noticeable difference in ASE values for model B. Both lighting simulation engines, Radiance [39] and Lumicept, are validated according to the CIE 171:2006 testing suite. However, as has been reported in [40], Radiance has an error in simulation of illuminance from direct sunlight. This exact light component is used for ASE calculation. Therefore, the ASE value calculated by DL-Light can be incorrect.

## 7. Conclusions

Nowadays, application of virtual sensing technology has become almost mandatory at an architectural project’s development stage. Daylight Autonomy metrics should be calculated multiple times during the project. Therefore, more efficient and accurate methods of DA metrics calculation are needed.

The Daylight Autonomy methods and algorithms elaborated during this study were implemented and added to the Lumicept software. Our algorithms work with arbitrary geometry and are not limited by any parametric models. Even with a blinds control algorithm, computational time is reduced to tens of minutes on a conventional computer, allowing the architect to constantly monitor compliance with standards during their project. DA metrics calculation for dozens of architectural models shows that our elaborated method is quite efficient and its accuracy sufficient for daylight analysis.

Our method was verified against a straightforward lighting simulation approach, Forward Monte Carlo ray tracing, which in turn has been validated with the CIE 171:2006 testing set. The verification shows good agreement; the difference in ASE and sDA metrics does not exceed 1–2%. Achieved simulation speed is higher than that of lighting simulation by FMCRT or other existing solutions based on the Radiance engine. The speed gain is more noticeable for more complex scenes and the computation time for them is several times less.

## Figures and Tables

**Table 1:** The sDA/ASE values for all verification scenes. No blinds control.

Verification Scene	sDA	ASE	Calculation Time
First scene (Figure 3)	100%	19.4%	0:09:27
Second scene (Figure 4)	90.4%	29.6%	0:09:44
Third scene (Figure 5)	95.7%	22.4%	0:10:58

**Table 2:** The sDA/ASE values for all verification scenes with blinds control.

Verification Scene	sDA	ASE	Calculation Time
First scene (Figure 3)	83.3%	19.4%	0:29:46
Second scene (Figure 4)	88.8%	29.6%	0:56:51
Third scene (Figure 5)	95.5%	22.4%	1:20:56

**Table 3:** Comparison of our method with FMCRT. The first verification scene (Figure 3).

Simulation Period	Our Method			FMCRT			Error	
	sDA	ASE	Calc. Time	sDA	ASE	Calc. Time	sDA	ASE
Winter (1–5 Jan)	72.2%	25.0%	0:06:18	72.2%	25.0%	0:25:58	0%	0%
Spring (1–5 Apr)	91.7%	75.0%	0:06:24	90.4%	75.0%	0:30:43	1.4%	0%
Summer (1–5 Jul)	100.0%	47.2%	0:06:28	100.0%	47.2%	0:33:25	0%	0%
Autumn (1–5 Oct)	91.7%	27.6%	0:06:23	91.7%	27.6%	0:29:21	0%	0%

**Table 4:** Comparison of our method with FMCRT. The second verification scene (Figure 4).

Simulation Period	Our Method			FMCRT			Error	
	sDA	ASE	Calc. Time	sDA	ASE	Calc. Time	sDA	ASE
Winter (1–5 Jan)	78.4%	30.4%	0:12:15	77.6%	30.4%	1:04:33	1.0%	0%
Spring (1–5 Apr)	88.8%	41.6%	0:09:17	88.8%	41.6%	1:10:46	0%	0%
Summer (1–5 Jul)	88.8%	26.4%	0:09:23	26.4%	88.8%	1:13:46	0%	0%
Autumn (1–5 Oct)	88.0%	19.2%	0:17:43	88.0%	19.2%	1:09:29	0%	0%

**Table 5:** Comparison of our method with FMCRT. The third verification scene (Figure 5).

Simulation Period	Our Method			FMCRT			Error	
	sDA	ASE	Calc. Time	sDA	ASE	Calc. Time	sDA	ASE
Winter (1–5 Jan)	95.2%	35.5%	0:18:42	95.5%	35.9%	0:58:22	0.4%	1.0%
Spring (1–5 Apr)	95.7%	21.3%	0:09:24	95.7%	21.3%	1:01:32	0%	0%
Summer (1–5 Jul)	95.2%	15.3%	0:15:06	95.0%	15.3%	1:05:11	0.2%	0%
Autumn (1–5 Oct)	95.7%	5.5%	0:09:20	95.9%	5.5%	0:58:47	0.2%	0%

**Table 6:** Comparison of calculation time for different simulation periods (from 1 to 5 days). The first verification scene (Figure 3).

Simulation Period	Our Method			FMCRT			Error	
	sDA	ASE	Calc. Time	sDA	ASE	Calc. Time	sDA	ASE
1 day	66.7%	25.0%	0:05:46	66.7%	25.0%	0:04:06	0%	0%
2 days	65.9%	25.0%	0:05:55	66.7%	25.0%	0:09:33	1.2%	0%
3 days	58.3%	25.0%	0:06:05	58.3%	25.0%	0:15:01	0%	0%
4 days	66.7%	25.0%	0:06:06	66.7%	25.0%	0:20:28	0%	0%
5 days	72.2%	25.0%	0:06:18	72.2%	25.0%	0:25:58	0%	0%

**Table 7:** Comparison of our method and DL-Light.

Model	Our Method			DL-Light		
	sDA	ASE	Calc. Time	sDA	ASE	Calc. Time
A	100%	8.6%	0:10:56	100%	8.7%	12 min
B	62.6%	3.1%	0:12:20	63.7%	4.6%	49 min

**Table 8:** Results of test 5.11 from CIE 171:2006. Points A–F on the wall (Figure 9).

CIE Points	A	B	C	D	E	F
		CIE sky model type 1				
CIE reference values	0.950	1.060	1.250	1.510	1.700	1.860
Lumicept	0.940	1.059	1.230	1.484	1.731	1.840
Error	1.1%	0.1%	1.6%	1.7%	−1.8%	1.1%
		CIE sky model type 8				
CIE reference values	0.950	1.060	1.420	2.130	2.640	2.810
Lumicept	0.951	1.062	1.41	2.095	2.646	2.791
Error	−0.1%	−0.2%	0.7%	1.6%	−0.2%	0.7%
		CIE sky model type 16				
CIE reference values	0.950	1.060	1.280	1.710	2.060	2.140
Lumicept	0.935	1.074	1.269	1.682	2.063	2.148
Error	1.6%	−1.3%	0.9%	1.6%	−0.1%	−0.4%

**Table 9:** Results of test 5.11 from CIE 171:2006. Points G–N on the floor (Figure 9).

CIE Points (Figure 9)	G	H	I	J	K	L	M	N
			CIE sky model type 1					
CIE reference values	0.870	1.310	2.020	3.200	5.070	7.640	9.330	5.090
Lumicept	0.857	1.312	2.021	3.203	5.012	7.637	9.279	5.031
Error	1.5%	−0.2%	−0.1%	−0.1%	1.1%	0.04%	0.5%	1.2%
			CIE sky model type 8					
CIE reference values	1.3	1.96	3.1	5.16	8.96	15.41	19.39	6.5
Lumicept	1.321	1.956	3.086	5.151	9.015	15.378	19.21	6.569
Error	−1.6%	0.2%	0.5%	0.2%	0.6%	0.2%	0.9%	−1.1%
			CIE sky model type 16					
CIE reference values	0.95	1.38	2.07	3.19	4.97	7.42	9.11	5.04
Lumicept	0.954	1.389	2.086	3.162	4.924	7.401	9.061	4.973
Error	−0.4%	−0.7%	−0.8%	0.9%	0.9%	0.3%	0.5%	1.3%

**Table 10:** Results of test 5.11 from CIE 171:2006. Points G’-N’ on the ceiling (Figure 9).

CIE Points (Figure 9)	G’	H’	I’	J’	K’	L’	M’	N’
			CIE sky model type 1					
CIE reference values	0.38	0.53	0.75	1.08	1.56	2.14	2.4	1.24
Lumicept	0.387	0.535	0.75	1.077	1.559	2.122	2.357	1.22
Error	−1.8%	−0.9%	0.0%	0.3%	0.1%	0.8%	1.8%	1.6%
			CIE sky model type 8					
CIE reference values	0.38	0.53	0.75	1.08	1.56	2.14	2.4	1.24
Lumicept	0.378	0.528	0.755	1.087	1.54	2.104	2.372	1.225
Error	0.5%	0.4%	−0.7%	−0.6%	1.3%	1.7%	1.2%	1.2%
			CIE sky model type 16					
CIE reference values	0.38	0.53	0.75	1.08	1.56	2.14	2.4	1.24
Lumicept	0.379	0.525	0.743	1.075	1.565	2.149	2.376	1.221
Error	0.3%	0.9%	0.9%	0.5%	−0.3%	−0.4%	1.0%	1.5%

## Data Availability

The data presented in this study are available within this paper. The used scenes in IOF format (Lumicept software) are available on request from the corresponding author.
